# Epicardial adipose tissue in patients with chronic obstructive pulmonary disease: systematic review with meta‑analysis and trial sequential analysis

**DOI:** 10.1186/s12890-023-02535-z

**Published:** 2023-07-03

**Authors:** Yi Lan, Qianli Ma, Guangming Luo, Heping Yang, Yingrui Li, Qiao Zhang

**Affiliations:** 1 Department of Pneumology, Songshan Hospital, Chongqing, China; 2 Department of Cardiology, The Second Affiliated Hospital of Chongqing Medical University, Chongqing, China

**Keywords:** Epicardial adipose tissue, Chronic obstructive pulmonary disease, Meta‑analysis

## Abstract

**Background:**

Limited data suggest that chronic obstructive pulmonary disease (COPD) patients have pathologic elevated epicardial adipose tissue (EAT), which is splanchnic fat tissue with anti-inflammatory properties and regulating free fatty acids functions. Therefore, there is a need for meta-analysis to explore the relationship between EAT and COPD.

**Methods:**

Online databases were systematically searched for studies about EAT in COPD patients published up to October 5th, 2022. The EAT data of the COPD patient group and the control group were included. Trial sequential analysis (TSA) and meta-analysis were applied to assess the difference in EAT between patients with and without COPD. TSA software and Stata 12.0 were used in all statistical analyses.

**Results:**

The final analysis included 5 studies (n = 596 patients). COPD patients had significantly more EAT than control subjects (SMD: 0.0.802; 95% CI: 0.231, 1.372; *P* = 0.006; TSA-adjusted 95% CI 1.20, 1.80; *P* < 0.0001). And higher CRP levels in COPD patients than non-COPD patients, whereas triglycerides and LDL were not significantly different between patients with and without COPD.

**Conclusion:**

EAT is abnormally elevated in COPD patients, which may be related to systemic inflammatory responses in COPD.

**PROSPERO number:**

CRD42021228273.

**Supplementary Information:**

The online version contains supplementary material available at 10.1186/s12890-023-02535-z.

## Background

As one of the most common respiratory diseases worldwide, chronic obstructive pulmonary disease (COPD) has higher morbidity and mortality in elderly people and, especially, in smokers over 40 years old [1]. The main pathological characteristic of COPD is irreversible limitation of airflow in the bronchial tubes, which is usually progressive and is related to abnormal inflammation caused by harmful particles or gases. Previous studies have suggested that systemic inflammation plays an important role in the pathological process of COPD [2, 3]. 70% of COPD patients suffer from systemic inflammation, while 16% suffer from persistent inflammation, which is manifested by elevated acute-phase proteins, chemokines, and circulating cytokines or abnormal cell numbers in the circulation [4, 5]. This kind of persistent inflammation can lead to a poor prognosis. Systemic inflammation makes COPD patients more likely to suffer from systemic comorbid conditions such as diabetes and cardiovascular disease (CVD) [6]. Recent studies suggested that COPD patients with systemic inflammation show 2-4-fold higher risks of cardiovascular disease, which is leading cause of deaths in COPD patients [7].

Epicardial fat that it is located on the surface of the myocardium is an extremely active organ [8]. Recent studies suggested that EAT is closely related to various systemic diseases, including atherosclerosis, metabolic syndrome, rheumatoid arthritis and systemic lupus erythematosus [9–12]. Under physiological conditions, EAT displays a protective effect on the heart via its anti-inflammatory properties and its function of regulating free fatty acids [13]. However, when EAT is abnormally increased in a pathological state, it can secrete a variety of inflammatory cytokines, including TNF-α, IL-8, IL-1 and IL-6, as well as extra fatty acids, which can cause systemic inflammation and abnormal serum levels of blood lipids (total cholesterol, triglycerides, HDL and LDL), finally leading to various systemic diseases [11, 12]. Considering the important role of systemic inflammation in the pathological process of COPD, EAT maybe is involved in the inflammatory process of COPD.

Recent studies detected higher EAT in patients with COPD, suggesting that EAT has the potential to become a novel marker of the risk of complications of COPD, such as cardiovascular disease and metabolic syndrome [14–18]. These studies’ small sample sizes and confounding factors lowered on the power of their results. The meta-analysis and Trial sequential analysis (TSA) were applied to assess the impact of EAT on COPD to explore whether EAT is associated with COPD.

## Methods

The meta-analysis strictly followed the PRISMA guidelines.

### Search strategy

In strict conformity with the PRISMA guidelines [19], the following terms were systematically searched for in the online database EMBASE, PubMed, Web of Science, and Cochrane Library: (Chronic obstructive pulmonary disease OR chronic obstructive lung disease OR chronic airflow limitation OR chronic obstructive airway disease OR chronic airway obstruction OR COPD) AND (epicardial adipose tissue OR subepicardial adipose tissue OR epicardial fat tissue OR subepicardial fat tissue). Only English studies and human studies were included. The databases were searched up to October 5th, 2022. The details of the search strategy are displayed in Table S1-4.

### Inclusion criteria

The analysis included studies that conformed to the following inclusion criteria: (1) patients with and without COPD were divided into a disease group and a control group; (2) the amount of EAT was measured by echocardiography, CMR or CT in both disease group and control groups; (3) the studies compared the differences in EAT amount between patients with and without COPD; and (4) the diagnostic criteria for COPD patients were reported in the study, as were the mean and standard deviation of the data and the sample size of each group. The details of the inclusion criteria are displayed in Table S5.

### Exclusion criteria

The analysis excluded studies with the following criteria: (1) nonclinical studies; (2) articles that did not provide sufficient information; and (3) repeat reports or secondary analyses of the same study population.

### Data extraction and quality evaluation

Data extraction and quality evaluation were independently completed by two reviewers (Yi Lan, Yingrui Li). These two reviewers discussed their assessments together to reach a consensus when they had differing opinions. EAT, EAT measurements, sample size, body mass index (BMI), age, blood lipids and inflammation indexes were extracted from the included studies. Then, the quality of the included studies was evaluated by applying the Newcastle–Ottawa Scale (NOS). There were three parts consisting of the scoring system, including selection of the included population, comparability between groups and measurement of exposure factors. The score of NOS ranges from 0 to 9. Better research quality can obtain higher scores (Table 1 and Table S6).

**Table 1:** Characteristics of Included Studies

Study	Country	Arms	N	Age(year)	BMI(Kg/m^2^)	Diagnosis	Total cholesterol, mg/dl	Triglycerides, mg/dl	HDL, mg/dl	LDL, mg/dl	C-reactive protein	EAT	Measurement tool	NOS Score
Zagaceta et al. 2013	Spain	COPD	171	59.0 ± 7.0	26.9 ± 4.8	GOLD	201.5 ± 45.8	/	51.0 ± 17.8	122.0 ± 46.4		143.7 ± 65.4 cm^3^	CT	9
		Non- COPD	70	57.0 ± 8.0	28.2 ± 5.4		202.0 ± 31.1	/	56.0 ± 15.6	123.0 ± 33.3		129.1 ± 58.9 cm^3^		
Higami et al. 2015	Japan	COPD	105	73.1 ± 7.5	23.1 ± 2.8	GOLD	/	/	/	/		13.1 ± 6.1 cm^2^	CT	9
		Non- COPD	26	67.0 ± 9.0	23.0 ± 3.3		/	/	/	/		10.9 ± 7.7 cm^2^		
Gaisl et al. 2015	Switzerland	COPD	81	64.3 ± 10.3	24.2 ± 5.8	GOLD	/	/	/	/	4 ± 4.5	132.6 ± 69.2 cm^3^	SPECT	7
		Non- COPD	81	64.3 ± 9.1	26.7 ± 4.9		/	/	/	/	4.1 ± 5.9	132.3 ± 90.2 cm^3^		
Kiraz et al. 2016	Turkey	COPD	157	69.6 ± 10.5	25.2 ± 4.6	Statement of ACP, ACCP, ATS, and ERS	177.9 ± 33.8	136.4 ± 50.2	40.3 ± 10.1	118.7 ± 26.6	0.8 ± 0.5	5.4 ± 1.6 mm	Echocardiography (End-systole parasternal long and short axis)	8
		Non- COPD	45	67.5 ± 4.1	24.3 ± 1.3		185.3 ± 14.5	143.4 ± 24.5	43.2 ± 6.2	122.9 ± 14.3	0.4 ± 0.2	4.1 ± 0.9 mm		
Demir et al. 2016	Turkey	COPD	51	66.0 ± 10.9	25.3 ± 3.8	GOLD	/	112.6 ± 49.5	45.2 ± 10.8	/	0.5 ± 0.3	6.1 ± 0.9 mm	Echocardiography (End-diastole parasternal long and short axis)	9
		COPD	31	66.1 ± 8.9	28.7 ± 4.7		/	181.7 ± 82.2	38.1 ± 10.5	/	0.6 ± 0.3	7.7 ± 1.8		
		Non- COPD	84	66.0 ± 9.6	29.7 ± 5.4		/	139.6 ± 62.5	49.1 ± 13.1	/	0.4 ± 0.2	4.8 ± 1.1 mm		

COPD, Chronic obstructive pulmonary disease; BMI, body mass index; LDL, low- density lipoprotein; HDL, high-density lipoprotein; EAT, epicardial adipose tissue; CT, computed tomography;SPECT, Single-photon emission computed tomography; GOLD, The Global Initiative for Chronic Obstructive Lung Disease; ACP, American College of Physicians; ACCP, American College of Chest Physicians; ATS, American Thoracic Society; ERS, European Respiratory Society

### Assessment of the quality of evidence

The overall quality of evidence was assessed using the Grading of Recommendations, Assessment, Development and Evaluations (GRADE) approach, which categorizes evidence into four levels of certainty: high, moderate, low, and very low. Given that all the studies included in the analysis were observational studies, the initial level of certainty was set as ‘Low’. Subsequently, the quality of evidence was downgraded based on five factors: (1) study limitations (limitations in the design and implementation of available studies suggesting high likelihood of bias); (2) indirectness (indirect population, intervention, control, outcomes); (3) inconsistency (Unexplained heterogeneity or inconsistency of results); (4) imprecision (wide confidence intervals); (5) publication bias. The quality of evidence can also be upgraded based on three factors: (1) large magnitude of effect; (2) adjusted for confounders (all plausible confounding would reduce a demonstrated effect or suggest a spurious effect when results show no effect); (3) Dose-response gradient.

### Data analysis

Stata 12.0 was used for all statistical analyses. The pooled standard mean difference (SMD) or weighted mean difference (WMD) with 95% CI was applied to calculate the overall effect size of cumulative data. All analyses in this analysis used a random effects model because of differences among the studies. All reported *P* values < 0.05 were regarded as statistically significant. Furthermore, χ^2^ and I^2^ statistics were applied to assess the statistical heterogeneity of the included studies. If I^2^ exceeded 50% and *P* was less than 0.1, the results were considered to have significant statistical heterogeneity [20]. Subgroup analysis of the EAT measurement was conducted to assess the effect of confounding factors on the meta-analysis results. To evaluate the reliability of the results, we assessed the effect of each study on the total merged results via sensitivity analysis. To assess the publication bias of the included studies, funnel plot analysis, Begg’s test and Egger’s test were conducted. *P* values < 0.10 were considered statistically significant [21]. Duval and Tweedie’s trim and fill method was used when publication bias occurred [22]. The details of the algorithms and scripts were shown in Supplementary material.

To obtain the total merged results, conventional meta-analyses conduct repeated significance tests on cumulative data, which increases the risk of type I errors. TSA applies the O’Brien-Fleming spending method to decrease the risk of type I errors and adjust the 95% CI of RR. The required information size (RIS) can be evaluated by TSA to examine whether the results have achieved the preset power level. TSA analysis can calculate whether more studies need to be carried out [23]. The repetitive significance testing of TSA was performed using a 5% risk of a type I error, a power of 80%, and an α-spending adjusted 95% CI.

## Results

### Characteristics of the studies

**Fig. 1:**
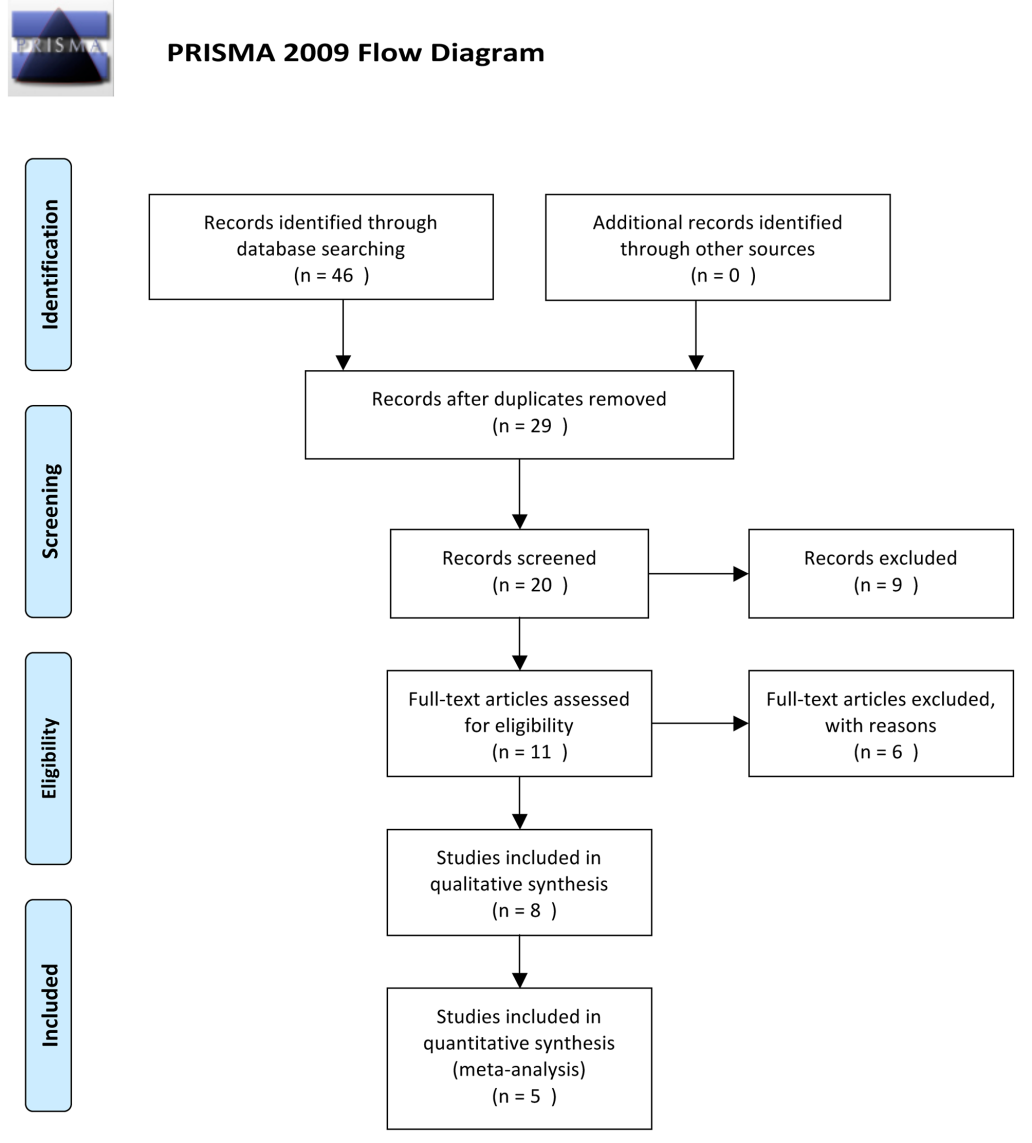
Flowchart for the process of study inclusion

The detailed search strategy yielded a total of 62 studies. Twenty-eight studies were rejected after excluding duplicate studies. Thirteen studies did not meet the inclusion criteria, and five studies that did not have insufficient data were excluded. Therefore, the final analysis included 5 studies (596 COPD patients and 306 healthy control patients) that measured the EAT of the COPD group and the control group [14–18] (Fig. 1).

Two studies measured the thickness of EAT by echocardiography [17, 18], 2 studies measured the volume or area of EAT by CT [14, 15], and 1 study measured the volume of EAT by single-photon-emission computed tomography (SPECT)[16]. To explore the sources of heterogeneity among the included studies, a subgroup analysis was performed based on different measurements of EAT. The results showed that heterogeneity in subgroups decreased significantly, suggesting that different EAT measurements might be the sources of the heterogeneity. The quality and features of these studies were evaluated by applying NOS. The amount of EAT, serum levels of blood lipids (total cholesterol, triglycerides, high-density lipoprotein (HDL), low-density lipoprotein (LDL)) and serum levels of C-reactive protein (CRP) were compared between patients with and without COPD. These characteristics are displayed in Table 1.

### Epicardial adipose tissue

**Fig. 2:**
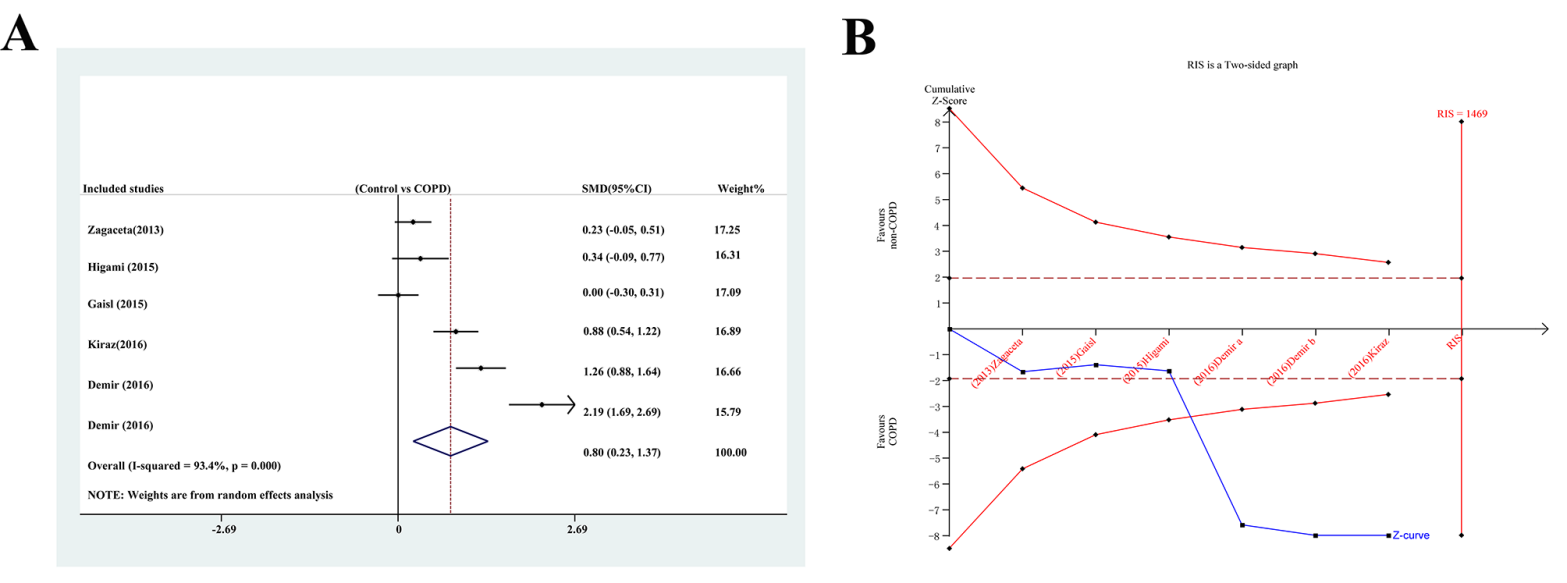
Meta-analysis and trial sequential analysis for EAT between patients with and without COPD. **(A)** Meta-analysis; **(B)** Trial sequential analysis

All studies reported on the EAT, and the results showed more EAT in the 596 patients with COPD than in the 306 non-COPD patients (SMD: 0.802; 95% CI: 0.231, 1.372; *P* = 0.006, Fig. 2a), with significant heterogeneity (I^2^ = 93.4%, *P* = 0.000). The TSA results showed that the futility boundary was crossed by the cumulative Z-curve, suggesting that the results were reliable (TSA adjusted 95% CI 1.20, 1.80; *P* < 0.0001, Fig. 2b).

**Fig. 3:**
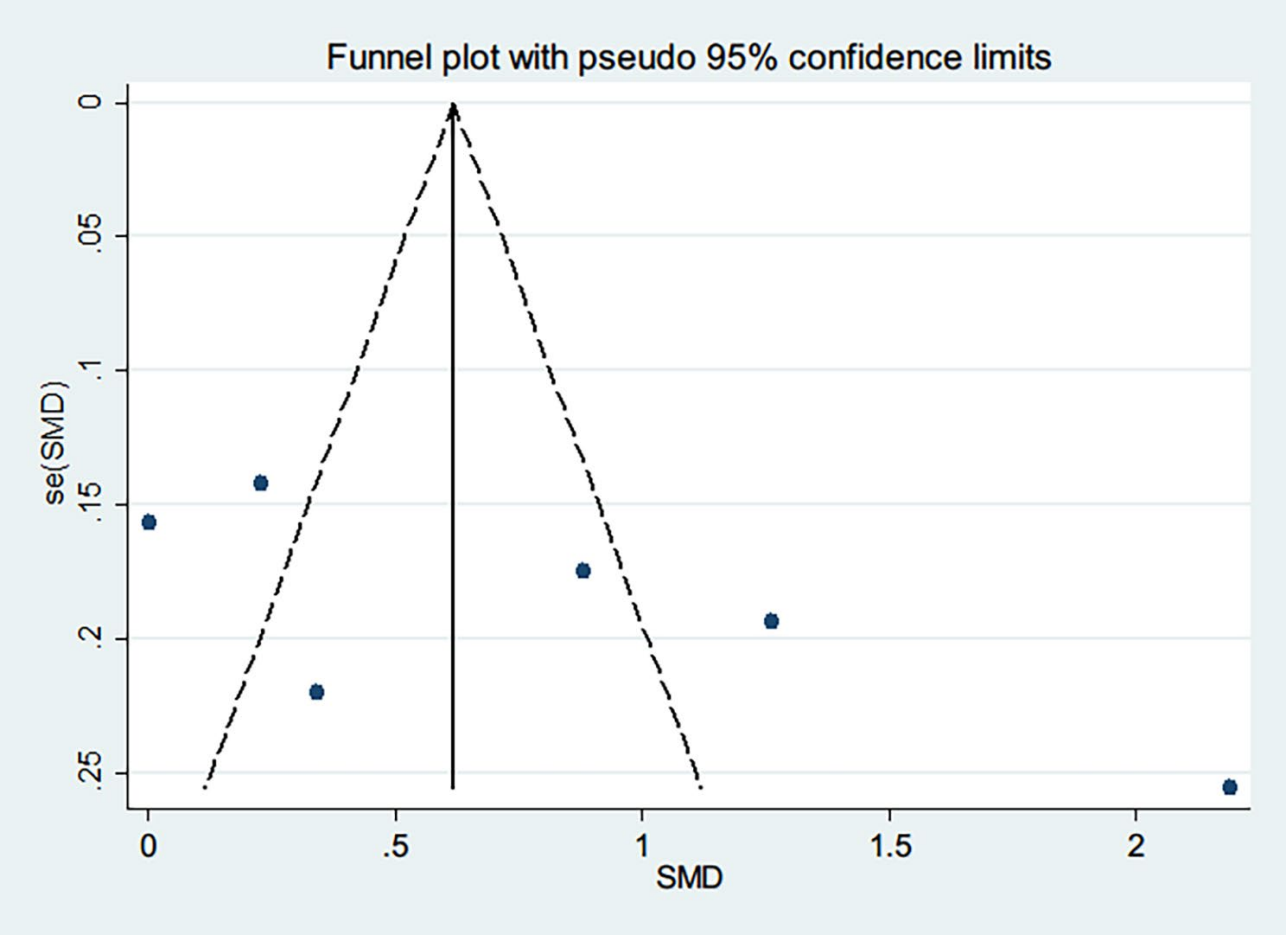
Funnel plot for publication bias assessment

The publication bias of the studies was evaluated by assessing the symmetry of funnel plots. The results showed an asymmetric distribution in the funnel plots (Fig. 3). To further evaluate publication bias, Begg’s test and Egger’s test were performed. Begg’s test (*P* = 0.133) and Egger’s test (*P* = 0.114) both revealed a low publication bias in the included studies.

Sensitivity analysis was performed by conducting an additional meta-analysis of the remaining studies after removing each study one by one, and the effect of every study deletion on the pooled SMD was evaluated. The results showed that none of the studies had an effect on the overall outcome (Table S7), indicating the stability of the meta-analysis results.

**Fig. 4:**
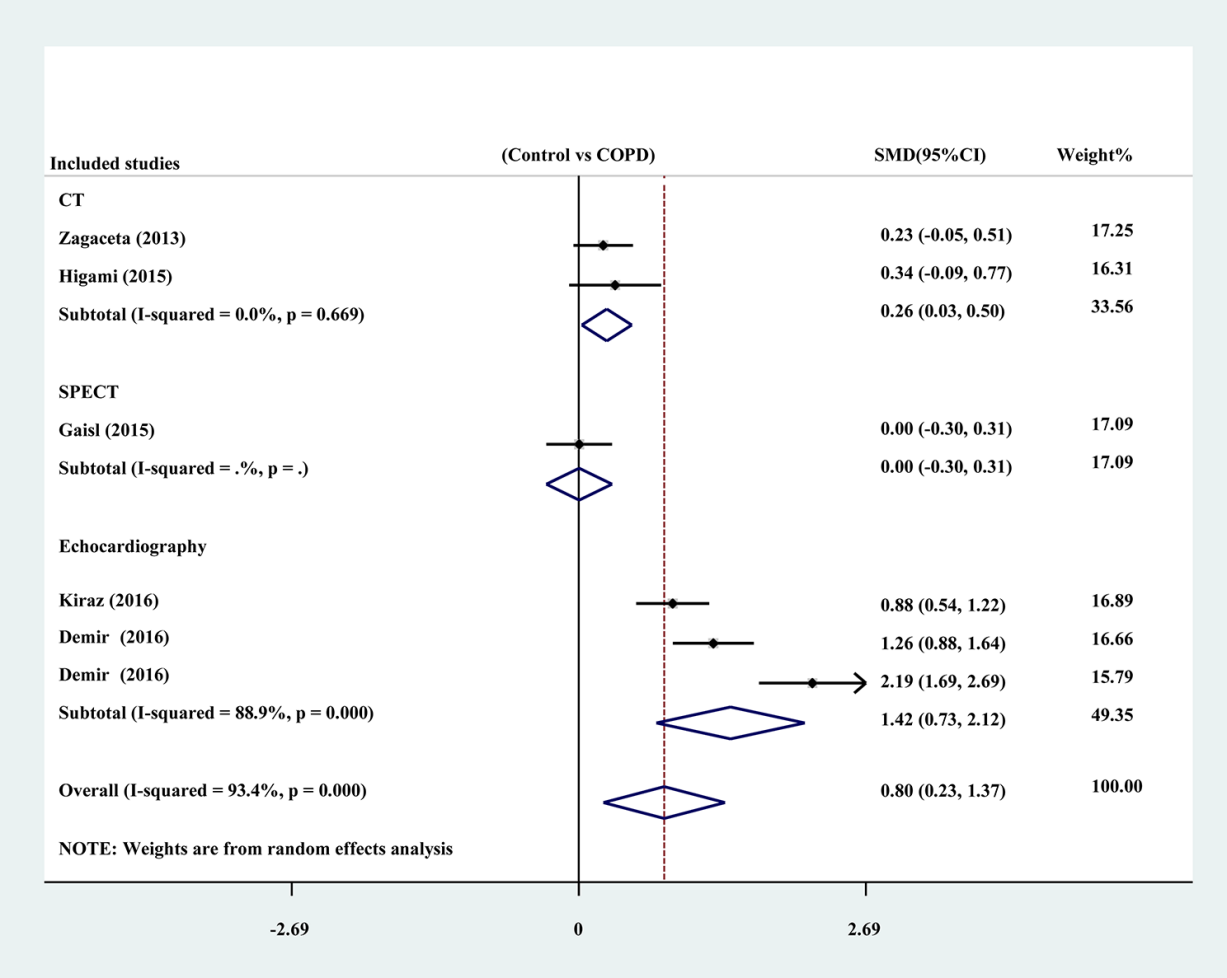
Forest plot of the subgroup analysis for EAT between patients with and without COPD by different measurements

To explore the potential sources of the significant heterogeneity in the analysis, a subgroup analysis was conducted. The included studies were divided into a CT measurement group, a SPECT measurement group and an echocardiography measurement group. The results showed that the heterogeneity among studies was greatly reduced in the CT group (n = 2) (SMD: 0.263; 95% CI: 0.028, 0.497; *P* = 0.028) (I^2^ = 0.0%, *P* = 0.669) and slightly reduced in the echocardiography group (n = 3) (SMD: 1.424; 95% CI: 0.729, 2.120; *P* = 0.000) (I^2^ = 88.9%, *P* = 0.000, Fig. 4). At the same time, the COPD patients in the CT group and echocardiography group had more EAT than the non-COPD patients.

### Blood lipids

**Fig. 5:**
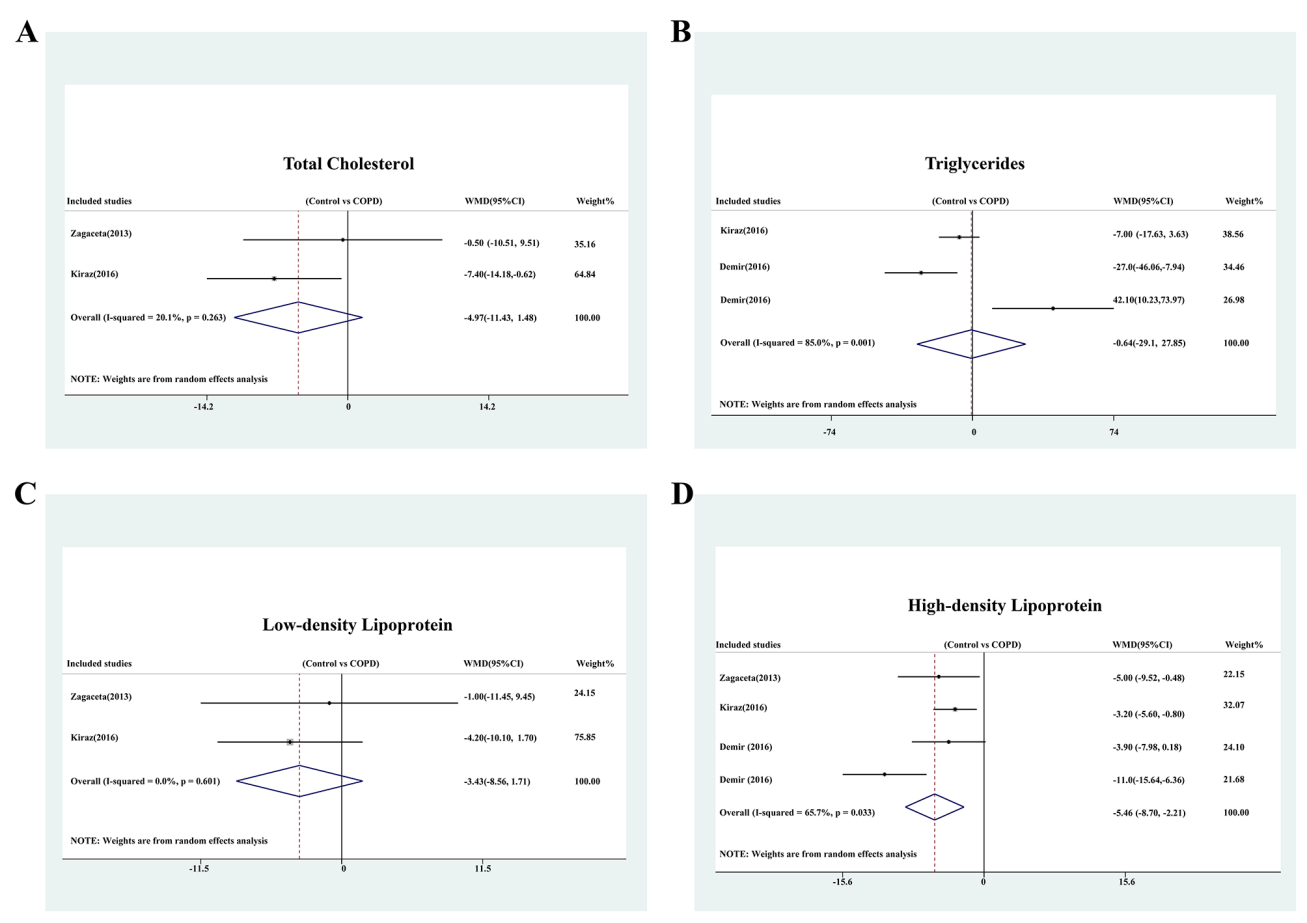
WMD in blood lipids between patients with and without COPD. **(A)** Forest plot of total cholesterol between patients with and without COPD; **(B)** Forest plot of triglycerides between patients with and without COPD; **(C)** Forest plot of LDL between patients with and without COPD; **(B)** Forest plot of HDL between patients with and without COPD.

Two studies reported total cholesterol, two studies reported triglycerides, three studies reported HDL, and two studies reported LDL. Total cholesterol (WMD: -4.974 mg/dl; 95% CI: -11.431, 1.483; *P* = 0.131) (I^2^ = 20.1%, *P* = 0.263, Fig. 5a), triglycerides (WMD: -0.643 mg/dl; 95% CI: -29.135, 27.849; *P* = 0.965) (I^2^ = 85.0%, *P* = 0.001, Fig. 5b) and LDL (WMD: -3.427 mg/dl; 95% CI: -8.563, 1.708; *P* = 0.191) (I^2^ = 0.0%, *P* = 0.601, Fig. 5c) displayed no significant difference between patients with and without COPD. However, COPD patients displayed much lower serum levels of HDL than non-COPD patients (WMD: -5.458 mg/dl; 95% CI: -8.703, -2.214; *P* = 0.001) (I^2^ = 65.7%, *P* = 0.033, Fig. 5d).

### C-reactive protein

**Fig. 6:**
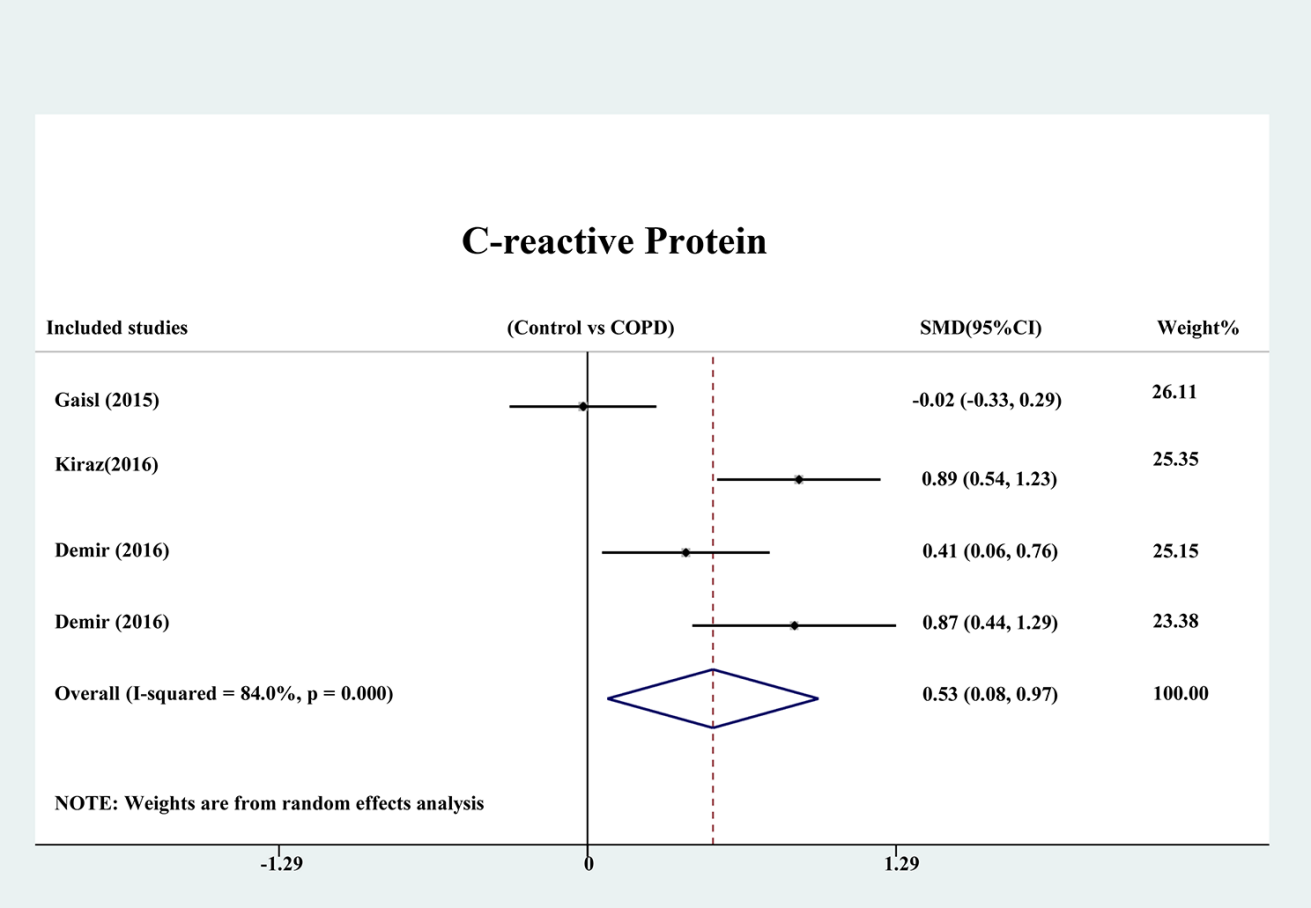
Meta-analysis of CRP between patients with and without COPD.

Three studies reported CRP, which is a valuable index of inflammation. Compared with non-COPD patients, COPD patients displayed significantly increased serum levels of CRP (SMD: 0.526; 95% CI: 0.084, 0.968; *P* = 0.020, Fig. 6), with great heterogeneity among the studies (I^2^ = 84.0%, *P* = 0.000).

### GRADE

The quality of evidence in the five studies detecting EAT was rated as very low according to the GRADE approach, attributable to the presence of wide confidence intervals observed in several studies (Table S8). As for LDL, HDL and total cholesterol, the quality of evidence was rated as low, with no downgrades or upgrades. Additionally, the quality of evidence for triglycerides and CRP were also rated as very low due to the wide confidence intervals reported (Table S8). Nevertheless, the analysis indicated a correlation between elevated CRP levels and increased EAT in COPD patients.

## Discussion

The results indicated that COPD is associated with more EAT, which was further confirmed by sensitivity analysis. Although the available samples did not reach the RIS by the TSA, the Z-curve intersected the boundary, indicating that the evidence was firm. Subgroup analysis suggested that patients with COPD showed abnormally increased EAT as detected by different measurements compared with patients without COPD. Total cholesterol, triglycerides and LDL were not significantly different between patients with and without COPD. However, COPD patients showed lower serum levels of HDL and higher CRP than non-COPD patients. These findings elucidated the potential implications of EAT in the inflammatory cascade and its capacity to assess the propensity for cardiovascular disease development in individuals with COPD.

Several studies on the relationship between EAT and COPD have been published in recent years, suggesting the increased amount of EAT was associated with risk of COPD and risk factors of cardiovascular disease in COPD patients [14, 15, 17]. In the research of Zagaceta and colleagues [14], the EAT of COPD patients increased significantly and was independently linked to risk factors that influenced the occurrence of future cardiovascular events, such as BMI, smoking history, and exercise capacity; at the same time, in the research of Higami et al., it was found that the thickness of the airway wall was independently correlated with EAT area in COPD patients [15]. Additionally, another study has elucidated a close association between EAT and the incidence of cardiovascular diseases, including coronary artery disease and atrial fibrillation [24]. The research, conducted by Ding et al., encompassed a cohort of 998 patients and revealed a positive association between EAT thickness and the occurrence of cardiovascular disease [25]. Consequently, these findings indicated that EAT can independently predict the risk of developing coronary artery disease in community-dwelling adults without a history of cardiovascular disease, as reported in population-based studies. Our study further displayed that COPD patients have increased EAT compared to non-COPD patients, which may be related to abnormal blood lipids and inflammatory processes in COPD patients. Hence, EAT might serve as an independent risk factor for the progression of cardiovascular disease in COPD patients to some extent, which holds significant implications for the management of COPD.

In line with the findings from past studies, the results of meta-analysis showed higher CRP levels in patients with COPD. COPD patients usually have an inflammatory pattern characterized by increased neutrophils in the trachea and increased macrophages, T lymphocytes and B lymphocytes in the circulation [26, 27]. CRP, an acute-phase protein, serves as a sensitive biomarker for systemic inflammation and tissue damage. Although CRP is not a specific inflammatory factor for COPD, it aids in clinical decision-making concerning the administration of antimicrobial agents for lower respiratory tract infections in COPD patients [28]. Studies have shown that CRP levels are higher during exacerbations of COPD compared to baseline levels, particularly in patients with COPD exacerbations caused by bacterial infections [29]. In patients with COPD, the elevated CRP was linked to an increased risk of the myocardial injury pattern on electrocardiogram (ECG) [30, 31], and CRP levels were also utilized to predict the incidence of cardiovascular events over 7 to 8 years of follow-up in the longitudinal Lung Health Study (LHS) [32]. Interestingly, recent studies suggested the closely association between EAT and inflammation. EAT that it is mainly composed of fat cells, immunocompetent cells, ganglia and interconnected nerve branches has not only unique anatomical features but also unique endocrine functions [8]. Previous studies indicated that EAT played a role in pathological conditions by secreting inflammatory cytokines, including leptin, IL-6, TNF-alpha and CRP [8, 33], which led to systemic inflammation and abnormal serum levels of blood lipids (total cholesterol, triglycerides, HDL and LDL) and finally caused various systemic diseases [11, 12]. The release of inflammatory cytokines can cause activation of immune cells and directly involve in airway remodeling in pathological process of COPD [34–36]. In addition to its role in pro-inflammatory cytokine secretion, EAT serves as an endocrine organ with metabolic activity, storing lipids and releasing numerous factors associated with atherosclerosis. Increased EAT has been associated with various cardiovascular metabolic diseases, including obesity, type 2 diabetes, metabolic syndrome, coronary artery disease, non-alcoholic fatty liver disease, and chronic kidney disease [37]. Therefore, compared to CRP, EAT exhibits superior potential in predicting the occurrence of metabolic syndrome and other comorbidities in COPD patients, thereby facilitating an enhanced patient prognosis.

Although the clinical studies suggested the important role of EAT in patients with COPD, the underlying mechanisms of the change in EAT observed in COPD patients have not been studied.

In addition, lipid metabolism disorders were also common in COPD, and HDL played an important role in both lipid metabolism and inflammation responses [38]. A recent study suggested HDL was an important indicator for predicting cardiovascular risk in COPD patients [39]. The present results also showed lower HDL levels in COPD patients, whereas total cholesterol, triglycerides and LDL were not significantly different between patients with and without COPD. Taken together, these results indicated that inflammation may be related to the increase in EAT in COPD patients.

EAT can be detected by several measurements, including echocardiography, CT and CMR. In recent years, CMR has been recommended to be regarded as the gold standard for detecting systemic adipose tissue because of its high resolution and good ability to quantify EAT [40]. However, CT or echocardiography is often used instead because of the high cost of CMR. CT also has higher resolution and the function of volume quantification, but patients are usually exposed to ionizing radiation and iodine contrast agent when they undergo CT examinations. Echocardiography is widely used, but its accuracy is lower than that of CMR and CT [41]. The subgroup analysis showed that the EAT of patients with COPD was higher in the CT group or echocardiography group, while there was no significant difference in the SPECT group. The small number of studies included in the SPECT group (n = 1) may have led to this result., and this may also be the resource of the high heterogeneity of results. The above results suggest that we could choose CT examinations or echocardiography measure the EAT in COPD patients in clinical practice.

The study conducted by Kunisaki et al. unveiled the impact of excessive ventilation, assessed via ultrasound echocardiography, on pulmonary hemodynamic alterations and subsequent cardiac function in COPD patients [42]. Similarly, Grau et al.‘s investigation of hospitalized patients with acute exacerbations of COPD (AECOPD) revealed the presence of pulmonary hypertension in all individuals examined [43]. The research showed that diminished lung elastic recoil due to emphysema leads to chest overinflation and increased intrathoracic pressure caused by airflow obstruction. This compromised lung elastic recoil, resulting in excessive ventilation, can disrupt the structures of the cardiac, systemic vascular, and pulmonary vascular systems, subsequently reducing venous return to the right and left ventricles. Additionally, excessive inflation, induced by positive end-expiratory pressure in COPD, can impede cardiac filling by diminishing venous return to the thorax and exerting direct pressure on the heart and pulmonary vasculature. Consequently, this process increases right ventricular end-diastolic stiffness and the pressure gradient in the pulmonary artery, ultimately impairing cardiac filling [43]. Nevertheless, it is noteworthy that changes in cardiac function in COPD patients do not significantly influence the composition or distribution of EAT, thereby having minimal impact on the accuracy of EAT measurements obtained through CT or echocardiography.

The meta-analysis suggested that COPD patients had more EAT than non-COPD patients, which is meaningful for clinical practice in the prediction in severity of COPD patients. Kalaycıoğlu et al. found a possible correlation between increased pulmonary artery pressure in COPD patients and thickening of EAT [44]. The endothelial dysfunction and inflammatory state in COPD patients may be related to increased pulmonary artery pressure, which could be attributed to EAT-induced endothelial dysfunction and the inflammatory state of endothelial cells. Additionally, the cytokines generated by EAT can directly enter the pulmonary circulation and increase inflammation in the pulmonary vascular bed. Consistent with previous findings, our results observed more EAT in COPD patients compared to non-COPD patients, although the exact reasons remain unclear. EAT also serves as a lipid storage depot and an endocrine organ that secretes hormones, as well as playing a role in the secretion of cytokines and chemokines in inflammatory tissues. Multiple studies have shown a correlation between increased EAT and various cardiovascular metabolic diseases, indicating that EAT can serve as a predictive factor for cardiovascular disease occurrence [45]. Groenewegen et al. demonstrated that the involvement of various inflammatory processes in COPD, which mediate the progression of diseases, while systemic inflammatory responses contribute to the development of diverse comorbidities [46]. Therefore, EAT may play a significant role in the inflammatory response process in COPD patients and can be employed for risk assessment and prediction of cardiovascular diseases in this population. Thus, our analysis supports the clinical significance of evaluating EAT volume and thickness in COPD patients to assess the inflammatory response and determine the risk of cardiovascular disease. However, Kiraz et al. observed a contradictory negative correlation between EAT thickness and the severity of COPD. The researchers suggested that this paradox might be related to the elevation of leptin in patients with coronary heart disease and COPD [17]. Further studies are needed to investigate the mechanisms underlying EAT increase in COPD and explore the potential correlations between COPD severity and EAT.

### Limitations

Several potential limitations should be noted. First, there were only 5 studies in the final analysis, and regression analysis could not be performed to find more potential factors that affected the results. Second, some data in the subgroup analysis only came from a few studies, so more studies should be conducted to support these results. Third, the great heterogeneity among studies could affect the reliability of the results. Even though several methods were used to explore the sources of heterogeneity and confirmed the stability of the results, all analyses could only partially explain the heterogeneity that existed in the analysis. Fourth, the analysis does not provide direct evidence to establish a causal relationship between changes in the volume and thickness of EAT and the presence of abnormal lipid levels and inflammation-related markers in COPD patients.

### Future directions

Our study revealed an increase in EAT in COPD patients, accompanied by elevated CRP levels and abnormalities in blood lipid profiles. Previous research suggested a potential correlation between the volume and thickness of EAT and CRP, indicating that EAT may play an important role in the inflammatory response observed in COPD patients. Furthermore, the increase in EAT might be associated with the occurrence of cardiovascular diseases in COPD patients. However, the exact contribution of EAT to the progression of COPD remains incompletely understood and necessitates further investigation. Our findings provide a theoretical basis for considering EAT as a potential novel biomarker in predicting the risk of cardiovascular disease or inflammation in COPD. Moreover, these results offer valuable insights for future research endeavours focusing on unravelling the mechanisms underlying the development of cardiovascular diseases and systemic inflammatory responses in COPD, with a specific focus on exploring the role of EAT.

## Conclusion

This meta-analysis indicated that patients with COPD have more EAT than control subjects, The results also showed that COPD patients had less HDL and more CRP.

## Electronic supplementary material

Below is the link to the electronic supplementary material.

Supplementary Material 1

## Data Availability

All data generated or analysed during this study are included in this published article [and its supplementary information files].

## Abbreviations

EAT: Epicardial adipose tissue
COPD: Chronic obstructive pulmonary disease
CVD: Cardiovascular disease
TSA: Trial sequential analysis
FFA: Free fatty acids
CMR: Cardiac magnetic resonance
CT: Computed tomography
SPECT: Single photon emission computed tomography
BMI: Body-Mass-Index
WMD: Weighted mean difference
SMD: Standard mean difference
RIS: Required information size
LDL: Low-density lipoprotein
HDL: High-density lipoprotein
CRP: C-reactive protein
NOS: Newcastle Ottawa scale
