# Supplementary material for: Epicardial adipose tissue in patients with chronic obstructive pulmonary disease: systematic review with meta‑analysis and trial sequential analysis
Source: BMC Pulm Med. 2023 Jul 3;23:241. doi: 10.1186/s12890-023-02535-z (PMC10318694; doi:10.1186/s12890-023-02535-z)
Supplement: Supplementary file 1 — Supplementary Material 1 [file 12890_2023_2535_MOESM1_ESM.docx]

**The Algorithms and Scripts**

Stata 12.0 was used for all statistical analyses. The overall effect size of the cumulative data was calculated using the pooled standard mean difference (SMD) or weighted mean difference (WMD) with a 95% confidence interval (CI). A random effects model using the DerSimonian & Laird method was applied due to observed differences among the included studies. Statistical significance was determined at a threshold of *P* < 0.05 for all reported results. Specifically, the "metan" script was used for random-effects meta-analysis, "metafunnel" and "metabias" scripts were employed for assessing publication bias through Begg's test, Egger's test, and generating a funnel plot, and sensitivity analysis was conducted using the "metatrim" script.

**Table S1. Search strategy of PubMed (https://pubmed.ncbi.nlm.nih.gov/)**

**Date of search: 05/10/2022**

| **#** | **Searches** | **Results** |
| --- | --- | --- |
| **1** | **"Epicardial adipose tissue" [Title/Abstract]** | **1465** |
| **2** | **"Epicardial adipose" [Title/Abstract]** | **1486** |
| **3** | **"Epicardial fat" [Title/Abstract]** | **1295** |
| **4** | **"Epicardial fat tissue" [Title/Abstract]** | **100** |
| **5** | **"Subepicardial adipose" [Title/Abstract]** | **22** |
| **6** | **"Subepicardial adipose tissue" [Title/Abstract]** | **22** |
| **7** | **"Subepicardial fat" [Title/Abstract]** | **26** |
| **8** | **"Subepicardial fat tissue" [Title/Abstract]** | **4** |
| **9** | **1 or 2 or 3 or 4 or 5 or 6 or 7 or 8** | **2458** |
| **10** | **"Chronic obstructive pulmonary disease" [Title/Abstract]** | **58190** |
| **11** | **"Pulmonary Disease, Chronic Obstructive"[Mesh]** | **64556** |
| **12** | **"COPD" [Title/Abstract]** | **55690** |
| **13** | **"Chronic obstructive lung disease" [Title/Abstract]** | **4515** |
| **14** | **"Chronic obstructive airway disease" [Title/Abstract]** | **314** |
| **15** | **"Chronic airflow limitation" [Title/Abstract]** | **360** |
| **16** | **"Chronic airway obstruction" [Title/Abstract]** | **357** |
| **17** | **10 or 11 or 12 or 13 or 14 or 15 or 16** | **100251** |
| **18** | **9 and 17** | **14** |

**Table S2. Search strategy of Web of science (https://clarivate.com/products/webofscience/)**

**Date of search: 05/10/2022**

| **#** | **Searches** | **Results** |
| --- | --- | --- |
| **1** | **"Epicardial adipose tissue" [TS]** | **2793** |
| **2** | **"Epicardial adipose" [TS]** | **2820** |
| **3** | **"Epicardial fat" [TS]** | **2120** |
| **4** | **"Epicardial fat tissue" [TS]** | **140** |
| **5** | **"Subepicardial adipose" [TS]** | **46** |
| **6** | **"Subepicardial adipose tissue" [TS]** | **46** |
| **7** | **"Subepicardial fat" [TS]** | **33** |
| **8** | **"Subepicardial fat tissue" [TS]** | **0** |
| **9** | **1 or 2 or 3 or 4 or 5 or 6 or 7 or 8** | **4306** |
| **10** | **"Chronic obstructive pulmonary disease" [TS]** | **89898** |
| **11** | **"Pulmonary Disease, Chronic Obstructive"[ TS]** | **49314** |
| **12** | **"COPD" [TS]** | **98685** |
| **13** | **"Chronic obstructive lung disease" [TS]** | **6040** |
| **14** | **"Chronic obstructive airway disease" [TS]** | **640** |
| **15** | **"Chronic airflow limitation" [TS]** | **464** |
| **16** | **"Chronic airway obstruction" [TS]** | **515** |
| **17** | **10 or 11 or 12 or 13 or 14 or 15 or 16** | **148230** |
| **18** | **9 and 17** | **23** |

**Table S3. Search strategy of Embase (https://www.embase.com)**

**Date of search: 05/10/2022**

| **#** | **Searches** | **Results** |
| --- | --- | --- |
| **1** | **"Epicardial adipose tissue" [ti, ab]** | **2441** |
| **2** | **"Epicardial adipose" [ti, ab]** | **2489** |
| **3** | **"Epicardial fat" [ti, ab]** | **2268** |
| **4** | **"Epicardial fat tissue" [ti, ab]** | **197** |
| **5** | **"Subepicardial adipose" [ti, ab]** | **29** |
| **6** | **"Subepicardial adipose tissue" [ti, ab]** | **28** |
| **7** | **"Subepicardial fat" [ti, ab]** | **29** |
| **8** | **"Subepicardial fat tissue" [ti, ab]** | **1** |
| **9** | **1 or 2 or 3 or 4 or 5 or 6 or 7 or 8** | **4245** |
| **10** | **"Chronic obstructive pulmonary disease" [ti, ab]** | **82258** |
| **11** | **"Pulmonary Disease, Chronic Obstructive"[ ti, ab]** | **33** |
| **12** | **"COPD" [ti, ab]** | **103318** |
| **13** | **"Chronic obstructive lung disease" [ti, ab]** | **6480** |
| **14** | **"Chronic obstructive airway disease" [ti, ab]** | **503** |
| **15** | **"Chronic airflow limitation" [ti, ab]** | **484** |
| **16** | **"Chronic airway obstruction" [ti, ab]** | **500** |
| **17** | **10 or 11 or 12 or 13 or 14 or 15 or 16** | **134869** |
| **18** | **9 and 17** | **23** |

**Table S4. Search strategy of Cochrane (https://www.cochranelibrary.com/library)**

**Date of search: 05/10/2022**

| **#** | **Searches** | **Results** |
| --- | --- | --- |
| **1** | **"Epicardial adipose tissue" [ti, ab, kw]** | **91** |
| **2** | **"Epicardial adipose" [TS]** | **91** |
| **3** | **"Epicardial fat" [TS]** | **137** |
| **4** | **"Epicardial fat tissue" [TS]** | **4** |
| **5** | **"Subepicardial adipose" [TS]** | **0** |
| **6** | **"Subepicardial adipose tissue" [TS]** | **0** |
| **7** | **"Subepicardial fat" [TS]** | **0** |
| **8** | **"Subepicardial fat tissue" [TS]** | **0** |
| **9** | **1 or 2 or 3 or 4 or 5 or 6 or 7 or 8** | **179** |
| **10** | **"Chronic obstructive pulmonary disease" [TS]** | **13438** |
| **11** | **"Pulmonary Disease, Chronic Obstructive"[ TS]** | **6238** |
| **12** | **"COPD" [TS]** | **18525** |
| **13** | **"Chronic obstructive lung disease" [TS]** | **7322** |
| **14** | **"Chronic obstructive airway disease" [TS]** | **79** |
| **15** | **"Chronic airflow limitation" [TS]** | **128** |
| **16** | **"Chronic airway obstruction" [TS]** | **57** |
| **17** | **10 or 11 or 12 or 13 or 14 or 15 or 16** | **23013** |
| **18** | **9 and 17** | **2** |

**Table S5. Eligibility criteria for study inclusion**

|  | **Inclusion criteria** | **Exclusion criteria** |
| --- | --- | --- |
| **Population** | Patients with and without COPD were divided into a disease group and a control group | Animals |
| **Intervention** | The amount of EAT was measured by echocardiography, CMR or CT in COPD patients | The amount of EAT has not been measured in COPD patients |
| **Comparator** | The amount of EAT was measured by echocardiography, CMR or CT in comparators | The amount of EAT has not been measured in comparators |
| **Outcomes** | The thickness or volume of EAT | Not reporting thickness or volume of EAT |
| **Study design** | Case-control study, one-to-one matched cohort study, randomized controlled trials | Nonclinical studies, cross-sectional, pre/post, case studies, case series |
| **Language** | English | Non-English publications |
| **Publication type** | Research article | Conference abstracts, protocols, commentaries, letter, repeat reports, secondary analyses of the same study population. |

**Table S6. Newcastle-Ottawa Scale (NOS) Evaluation of Study Quality**

| **STUDY** | **SELECTION** | **COMPARABILITY** | **EXPOSURE FACTORS** |
| --- | --- | --- | --- |
| **Zagaceta et al.** | ******** | ****** | ******* |
| **Higami et al.** | ******** | ****** | ******* |
| **Gaisl et al.** | ******* | ****** | ****** |
| **Kiraz et al.** | ******** | ****** | ****** |
| **Demir et al.** | ******** | ****** | ******* |

**The Newcastle-Ottawa Scale (NOS) evaluates the included studies based on selection, comparability and outcome. The maximum score for each criteria is 4, 2 and 3, respectively, with the maximum total score equaling 9.**

**Table S7. Sensitivity analysis displaying pooled SMD and 95% confidence intervals with systematic exclusion of individual studies.**

| **Excluded study** | **Pooled SMD** | **Lower 95% CI** | **Upper 95% CI** | ***I*^2^** | **p-value** |
| --- | --- | --- | --- | --- | --- |
| **Zagaceta et al.** | **0.620** | **0.048** | **1.191** | **90.0%** | **0.033** |
| **Higami et al.** | **0.894** | **0.225** | **1.563** | **94.6%** | **0.009** |
| **Gaisl et al.** | **0.966** | **0.341** | **1.591** | **92.9%** | **0.002** |
| **Kiraz et al.** | **0.789** | **0.094** | **1.484** | **94.5%** | **0.026** |
| **Demir et al.** | **0.710** | **0.079** | **1.341** | **93.6%** | **0.027** |
| **Demir et al.** | **0.538** | **0.091** | **0.984** | **88.3%** | **0.018** |

**Table S8. Evaluation of the quality of evidence using GRADE framework.**

|  | **Possible downgrades** |  |  |  |  | **Possible upgrades** |  |  | **Overall quality** |
| --- | --- | --- | --- | --- | --- | --- | --- | --- | --- |
| **Outcomes** | **Study limitations** | **Indirectness** | **Inconsistency** | **Imprecision** | **Publication bias** | **Large magnitude of effect** | **Adjusted for confounders** | **Dose-response gradient** |  |
| **EAT** | **No** | **No** | **No** | **Yes** | **No** | **No** | **No** | **No** | **Very Low** |
| **LDL** | **No** | **No** | **No** | **No** | **No** | **No** | **No** | **No** | **Low** |
| **HDL** | **No** | **No** | **No** | **No** | **No** | **No** | **No** | **No** | **Low** |
| **Total cholesterol** | **No** | **No** | **No** | **No** | **No** | **No** | **No** | **No** | **Low** |
| **Triglycerides** | **No** | **No** | **No** | **Yes** | **No** | **No** | **No** | **No** | **Very Low** |
| **CRP** | **No** | **No** | **No** | **Yes** | **No** | **No** | **No** | **No** | **Very Low** |
